# Multiple Objects Fusion Tracker Using a Matching Network for Adaptively Represented Instance Pairs

**DOI:** 10.3390/s17040883

**Published:** 2017-04-18

**Authors:** Sang-Il Oh, Hang-Bong Kang

**Affiliations:** Department of Media Engineering, Catholic University of Korea, 43-1, Yeoggok 2-dong, Wonmmi-gu, Bucheon-si, Gyeonggi-do 14662, Korea; nicolas0@catholic.ac.kr

**Keywords:** multiple objects tracking, deep learning, multiple sensor fusion, LIDAR, CCD

## Abstract

Multiple-object tracking is affected by various sources of distortion, such as occlusion, illumination variations and motion changes. Overcoming these distortions by tracking on RGB frames, such as shifting, has limitations because of material distortions caused by RGB frames. To overcome these distortions, we propose a multiple-object fusion tracker (MOFT), which uses a combination of 3D point clouds and corresponding RGB frames. The MOFT uses a matching function initialized on large-scale external sequences to determine which candidates in the current frame match with the target object in the previous frame. After conducting tracking on a few frames, the initialized matching function is fine-tuned according to the appearance models of target objects. The fine-tuning process of the matching function is constructed as a structured form with diverse matching function branches. In general multiple object tracking situations, scale variations for a scene occur depending on the distance between the target objects and the sensors. If the target objects in various scales are equally represented with the same strategy, information losses will occur for any representation of the target objects. In this paper, the output map of the convolutional layer obtained from a pre-trained convolutional neural network is used to adaptively represent instances without information loss. In addition, MOFT fuses the tracking results obtained from each modality at the decision level to compensate the tracking failures of each modality using basic belief assignment, rather than fusing modalities by selectively using the features of each modality. Experimental results indicate that the proposed tracker provides state-of-the-art performance considering multiple objects tracking (MOT) and KITTIbenchmarks.

## 1. Introduction

Object tracking is an important task in various research areas, such as surveillance, sports analysis, human-computer interaction and autonomous driving systems. As a result, various forms of tracking are being actively researched; these include multiple objects tracking (MOT), tracking using multiple sensors and model-free tracking. In this paper, we propose a new multiple-object tracker that uses multi-sensor modality. The main objective of MOT is to estimate the states of target objects from given frames in a video sequence. However, despite much success, MOT techniques still face various challenges caused by illumination and scale changes, occlusion and other disturbance factors.

One way to handle distortions is to model them into trackers a priori. Affine transformation [1], illumination invariance [2] and occlusion detection [3,4] have been widely applied to trackers to deal with disturbances. While trackers in which distortion handlers are embedded are able to overcome a specific disturbing factor, tracking may fail when other distortions are introduced. Another way to maintain tracking performance despite distortions is to adaptively train the appearance (and/or motion) model of the object tracker online. Although the appearance models are adaptively updated, dynamically-changed appearances can be missed when temporary changed appearances are included in the newly-updated appearance models.

In addition, when trackers are only generated on RGB frames, shifting (in the case of shifting from the bounding box of a target object at time *k* to a similar object at a time k+1, owing to the similarity in terms of shape, color and other factors between objects) of target boxes occurs easily. To compensate for tracking failures resulting from various disturbance factors in each modality, tracking using multi-sensor fusion has been researched [5,6,7,8,9,10]. Tracking failures on RGB frames can be compensated by using depth information from 3D point clouds and stereovision sensors. However, trackers function properly only when all of the modalities are operated without any noise, i.e., information conflicts between modalities have a negative effect on the accuracy of trackers.

**Figure 1:**
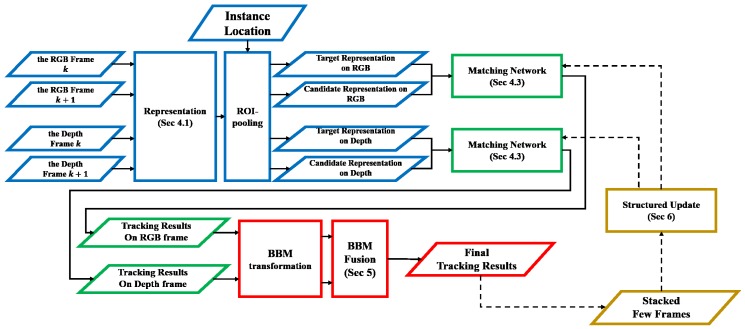
The overall architecture of MOFT. The blue box: representation (Section 4.1 and Section 4.2). The green box: matching between target objects and candidates (Section 4.3). The yellow box: structured fine-tuning (Section 6). The red box: fusion of tracking results (Section 5).

In this work, we propose a new tracker that improves the performance of MOT. The proposed tracker, called the multiple object fusion tracker (MOFT), learns a generic matching function without modeling for specific disturbing factors on multiple modalities. Figure 1 shows the architecture of the MOFT. The tracker takes paired representations consisting of a target object at frame *k* and a target candidate at frame k+1 as inputs (blue box in Figure 1). To represent instances, we adaptively use the output of a convolutional layer as the representations of the instance according to its scale. Then, we feed paired representations into a matching network to extract the matching score (green box in Figure 1). The matching network is initialized on external sequences using a convolutional neural network (CNN) comprising two sub-networks that share weights. To improve the tracking performance, we combine independent tracking results on each modality to compensate the failures occurring in each modality (red box in Figure 1). Finally, we fine-tune the matching network initialized on the external video sequences by using few stacked tracking results with structured appearance models (yellow box in Figure 1).

CNNs have previously been employed in trackers [11,12]. Those trackers used only the fixed outputs of layers from CNN architectures as a representation of target objects. However, because target objects have different scale levels, information loss can occur [13]. To solve this problem, we adaptively select the convolutional layer from a pre-trained CNN according to the scales of the target objects as a representation. We train a matching function that can be well fitted to generic distortions without adaptively updating models during testing, while still achieving state-of-the-art performance. To achieve this goal, large-scale external video sequences are used to initialize a matching function. For the tracker, we train a CNN comprising two sub-networks sharing weights, called the matching network, in an offline manner. During testing, the matching scores between a set of target objects in frames *k* and k+1 are computed using the learned matching network with frozen weights. At this point, the training datasets used to initialize the matching network are not overlapped with the sequences for testing because our aim is to build a generic matching function that can be applied to any unseen object.

We use CNNs that are pre-trained on large-scale annotated datasets. Although many kinds of pre-trained CNNs for representing RGB images are available, to the best of our knowledge, no CNNs pre-trained on depth data exist. To overcome this problem, we apply supervision transfer [14] to transfer from learned representation on large-scale annotated data (source data) to another modality (target data) for tracking. Supervision transfer is generated on paired data modalities and facilitates the representation of data without annotations.

To compensate the limitations of tracking in RGB frames, in this paper, we propose a fusion tracker that combines the tracking results of RGB frames and depth frames. In particular, to solve the aforementioned problems besetting feature-fusion tracking schemes, we fuse tracking results at the decision level. In the proposed decision-level-fusion scheme, fusion is performed when MOT is completed on each modality using basic belief masses (BBMs).

For the first few frames, tracking is performed by using the initialized matching network. After performing tracking on a few frames, the initialized matching network is fine-tuned based on the weighted average of the matching score to update the target appearance models. At this point, fine-tuning is not performed on the same matching network iteratively. According to the matching score extracted from the matching network, we construct a structured path for fine-tuning. Targets that have a similar appearance model activate the same path of fine-tuned matching networks while the targets’ difference to previous paths is updated on a newly-created node. The proposed MOFT performs well without this update procedure, but the MOFT with structured target appearance models can more precisely track temporally-changed target states.

The contributions of this work are as follows: (1) matching function learning that can be applied without adaptive updating from large-scale external video sequences; (2) a proposed fusion tracker that fuses at the decision level; and (3) the structured fine-tuning strategy for the matching function. Our proposed tracker achieved state-of-the-art tracking performance on published object tracking benchmark datasets. After the optimization of our code, we plan to submit it to benchmark competitions.

## 2. Related Work

Deep learning tracking: Several trackers have used neural networks with training models trained online for tracking [15,16,17,18,19,20]. Among them, the trackers proposed by Danelljan et al. [15] and Nam and Han [16] exhibit state-of-the-art performance for the tracking task. However, because online neural network training is very slow, these trackers are very slow. Recently, several trackers that use a matching function trained offline using a CNN architecture have been presented. Siamese instance search for tracking (SINT) [11] is a simple tracker that matches the initial target object in the first frame with candidates in the current frame using a Siamese network. Similarly, generic object tracking using regression networks (GOTURN) [12] trains a neural network offline and can track objects at 100 fps at test time. GOTURN reduces the search candidates using a search strategy in which the locations of moving objects are slowly changed from frame *k* to frame k+1. Previously-proposed trackers that use CNNs in an offline manner represent instances using a fixed number of layers. However, because instances have different scale levels, representing them using fixed layers can result in information loss.

Fusion of multiple modalities for vision tasks: In many applications, multiple modalities have been fused to completely generate their tasks [21,22,23,24,25,26]. In particular, tracking results with disturbances occurring in RGB frames have been compensated by combining depth information extracted from laser scanners, stereo vision and RGB-D sensors. For robotics systems, many kinds of modalities are fused to track moving objects. In sports analysis, the moving objects are tracked from sets of calibrated cameras to precisely localize the objects [21,22]. The depth data from stereo vision sensors, Kinect and 3D LIDAR are often fused with RGB images for dynamic environments [23,24,25]. Mertz et al. [27] proposed a detector and tracker that combine single-layer and multi-layer (LIDAR) laser scanners. Cho et al. [24] proposed a tracker that uses radar, LIDAR and CCD sensor data in which their features are fused. Aufere et al. [28] presented a fusion approach for avoiding collisions by tracking through the use of CCD and LIDARs. In a benchmark presented by Sturm et al. [29], there are various trackers using RGB-D data for visual odometry and SLAM tasks. Teutsch et al. [30] proposed an object detector that used the long wavelength infrared camera to detect low resolution objects. Kummerle et al. [31] fused the multiple infrared spectrum images to detect and track multiple humans from a bird’s eye view. Gozalez et al. [32] presented an object detector, which leverages multiple cues from the multi-sensor modality for driving situations. They claimed that each modality has different views and features, and these aspects can increase the detection accuracy by aggregating them. The fusion trackers cited above separately extract features from each modality in order to fuse them and required equally well-captured multi-sensor data for generating them with accurate performances. However, because they cannot consider beliefs about sensor measurements, these trackers are significantly influenced by noise from each modality.

## 3. Problem Definition and Pre-Requisites

In this section, we describe the traditional issues that the proposed tracking method addresses. Figure 1 shows the overview of the proposed method. Suppose there exist RGB frames and 3D point clouds, which should be pre-calibrated and synchronized. The proposed tracker can be independently applied on RGB frames or 3D point clouds without following the fusion procedure; however, we fuse both data to compensate for each other’s tracking failures. Because the coordinate systems of two modalities differ, the coordinate systems between two different modalities should be homogenized to find the associations when the tracking results are fused at the decision level. Therefore, the proposed MOFT is generated on image coordinates for both RGB frames and 3D point clouds. Pre-defined algorithm settings for each step will be discussed in Section 7.2. We also evaluate tracking performances when the proposed tracker is independently applied on each modality in Section 7.3. Note that the maximum size of an object to be tracked is set to the image size because the tracking is performed on image coordinates for both modalities. To track objects on 3D point clouds in image coordinates, we employ a transformation method [33], which transforms 3D point clouds to a dense depth map by up-sampling the point clouds.

The representation step means representing object instances (e.g., the bounding box of each object) using a feature extraction method. Each instance $x_{i}$ has state $x_{i}=\left[x,y,w,h\right]$ where *x* and *y* denote the left-top location, and *w* and *h* are the width and height, respectively. In this paper, we adaptively use an output of the VGG-16 architecture [34] pre-trained on the ILSVRC2012 as a representation of each object instance according to size. To identify the size of each instance, we use the scale-dependent pooling (SDP) [35]. By doing this, the instances with different sizes can be suitably represented without any information loss. To identify the matched pairs between given representations of target object instances at time *k* ($X^{k}=\left\{x_{i,i=1,\ldots ,n}^{k}\right\}$) and representations of candidate instances at time k+1 ($X^{k+1}=\left\{x_{j,j=1,\ldots ,m}^{k+1}\right\}$), we train a matching network. Before finding matched pairs, the candidates at time k+1 should be set. The MOFT uses both traditional candidate selection methods for tracking, which include candidate sampling [11] and the object detector [36]. Because directly accepting the bounding boxes of instances extracted from the candidate sampling method or object detector can increase localization error, we use a bounding box regressor [37], which regresses center coordinates of the box and their width and height.

## 4. Matching Network

We propose a matching function that can track objects when various disturbing factors are introduced into the scenes.

### 4.1. Target Representation

**Figure 2:**
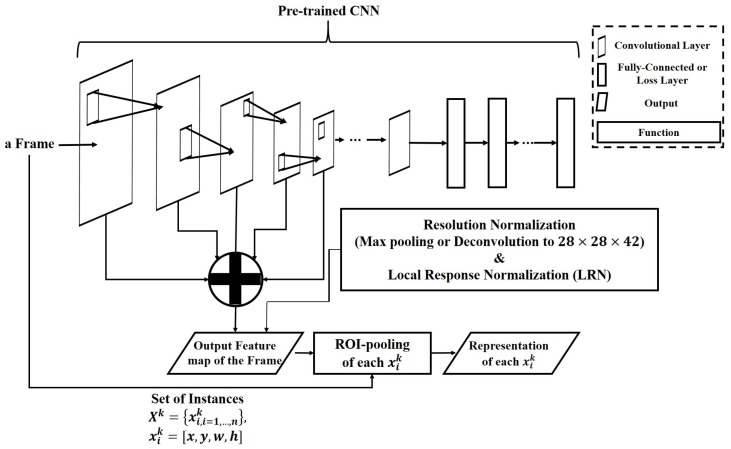
Architecture for representing instances.

Target objects contain large variations in their scales according to disturbance factors, such as pose change and movement states. If the instances that have a low resolution (i.e., small scale) are passed to subsequent layers, fine features may be gradually ignored owing to operations such as convolution and pooling. To suitably represent instances according to their scales, we first classify scales into a sampled range (scale-level ranging is discussed in Section 4.1). If the scale of instances is small, the earlier output of the convolutional layer is extracted as a representation, while the latter output of the convolutional layer is extracted as a representation. Figure 2 shows the architecture of the proposed representation method.

Feeding entire instances into the pre-trained network can result in high computational cost. In this work, we apply ROI pooling [38] to frame representation to reduce the computational cost. First, we extract all of the outputs of the convolutional layers of a frame from the pre-trained CNN. Then, the representation of each instance is pooled from the output map of its corresponding frame according to its scale level using ROI pooling. Because the sizes of the outputs from each convolutional layer vary, we sample them individually by applying different sampling layers to feed them into the matching function. Max pooling is applied to sub-sample the represented instances with sizes greater than the input size of the matching network. A deconvolutional operation is also used to up-sample represented instances that are smaller than the input size of the matching network. Finally, local response normalization (LRN) [39] is used to normalize all of the representations.

### 4.2. Representation of the Depth Frame

To represent instances of depth data that have no pre-trained CNNs on large-scale annotated dataset, we employ supervision transfer [14].

Let $\Phi =\{\phi_{i},i\in \left[1,\ldots ,\kappa \right]\}$ and $\Psi =\{\psi_{i},i\in \left[1,\ldots ,\kappa \right]\}$ be layered representations of RGB and depth frame, respectively, where *i* denotes the number of layers. The main task of supervision transfer is to effectively learn the weight parameters $W_{\left[1,\ldots ,\kappa \right]}=\left\{w_{i},i\in \left[1,\ldots ,\kappa \right]\right\}$ to represent unannotated depth images from a fixed CNN architecture. Supervision transfer proceeds by measuring the similarity between the representations of both modalities by using a loss function *f* (in this work, $L_{2}$ distance is used for the loss function). The similarity can be measured as follows:

(1) $$arg\min_{W^{\kappa }}\sum_{i=1}^{\kappa }f\left(t\left(\psi_{i}\right)w_{i},\phi_{i}\right),$$

where t(•) is the transformation function for embedding $\psi_{i}$ into the same dimension of $\phi_{i}$. $w_{i}$ denotes the learned weight parameter. In this work, if depth images are obtained from 3D point clouds, the up-sampling method [33] is used for the transformation function.

### 4.3. Architecture of the Matching Network

**Figure 3:**
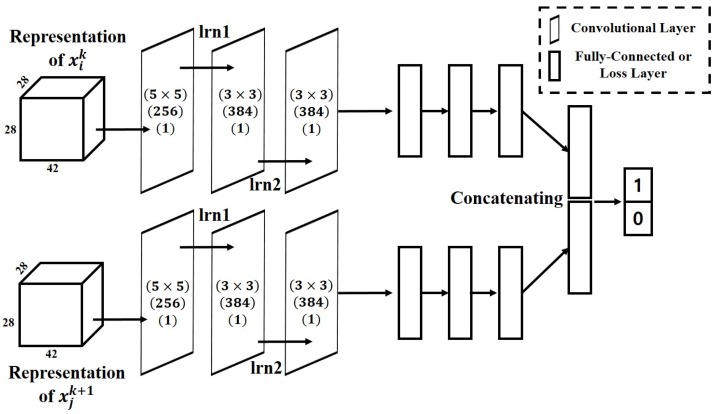
Proposed matching network to learn the matching function. The numbers in brackets on convolutional layers are the kernel size, number of outputs and stride sizes, from the top. $x_{i}^{k}$ and $x_{j}^{k+1}$ are the representation of the *i*-th target object in frame *k* and the *j*-th candidate in frame k+1.

To train a generic matching function that can be generated on sequences that include disturbing factors, we employ a CNN architecture comprising two sub-networks sharing weights as a matching network. The proposed matching network is shown in Figure 3. To construct each paired input $\left(x_{i}^{k},x_{j}^{k+1}\right)$, representations (Section 4.1 and Section 4.2) of target object $x_{i}^{k}$ and candidates $x_{j}^{k+1}$ are separately fed into two identical sub-networks that are in the form of a CNN. For the proposed matching network, the sub-network comprises three convolutional layers and two fully-connected layers.

In traditional CNN architectures, strong values in the local neighborhood can only be activated to be fed into subsequent layers when max pooling is applied on inputs, i.e., the spatial resolutions of activated values are substantially reduced. While one benefit of max pooling is invariance to local deformations, maintaining the small appearance changes of the target objects over time is more important for the MOT task. Therefore, the max pooling layers are not included in the proposed sub-network architecture.

Finally, the outputs of the last fully-connected layer in each sub-network are concatenated and fed into a two-way Softmax layer. The Softmax layer is used to determine the matching between $x_{i}^{k}$ and $x_{j}^{k+1}$. In our work, Classes 1 and 0 denote the matching (positive) and mismatching (negative), respectively.

## 5. Fusion Tracker

The proposed tracker is independently generated from represented instances, both RGB and depth frames. We obtain more accurate tracking results by fusing the tracking results generated from each modality at the decision level than using only the tracker-generated RGB frames.

In this section, we present a method of assessing the discounting factors to be applied to the tracking results from each modality using the basic belief assignment (BBA). The discounting factor α indicates the degree of trust to apply to the tracking results.

### 5.1. Basic Belief Assignment

The BBA is based on the Dempster–Shafer theory (DST) [40,41]. The DST is a generalization of the Bayesian inference. The frame of discernment $\Omega =\{a_{i,i=1,\ldots ,n}\}$ is the set of elementary hypotheses in the DST. Elementary $a_{i}$ are disjointed with each other while they cover the complete event space. The mass function *m* of the BBA to map the power set $2^{\Omega }$ to [0,1] is defined as follows:

(2) $$\sum_{A\subseteq \Theta }m\left(A\right)=1,m\left(\emptyset \right)=0,$$

where m(A) represents the certainty for proposition *A* of belief committed to the subset. All BBAs are assigned as probability functions, i.e., they are measurements of certainty.

The Dempster–Shafer combination rule is applied to combine two different BBAs, $m_{1}$ and $m_{2}$, as follows:

(3) $$m_{1⊕2}\left(A\right)=m_{1}\left(A\right)⊕m_{2}\left(A\right)=\frac{\sum_{X\cap Y=A}m_{1}\left(X\right)m_{2}\left(Y\right)}{1-\sum_{X\cap Y=\emptyset }m_{1}\left(X\right)m_{2}\left(Y\right)},\forall A\in 2^{\Omega }.$$

The BBA consists of three components: belief $bel_{m}\left(A\right)=\sum_{B\subseteq A,B\neq \emptyset }m\left(B\right)$, plausibility $pl_{m}\left(A\right)=\sum_{B\cap A\neq \emptyset }m\left(B\right)$ and uncertainty $U_{m}\left(A\right)=Pl_{m}\left(A\right)-Bel_{m}\left(A\right)$. The degree of $bel_{m}\left(A\right)$ is set as the support of the BBA *m* for *A*. The degree of $pl_{m}\left(A\right)$ is assigned by the sum of all BBAs of *m* that do not contradict *A*.

Belief $bel_{m}$ and plausibility $pl_{m}$ can be construed as pessimistic and optimistic guesses that ignore the additional information for uncertainty if a decision is required. The pignistic transformation [40] can incorporate this additional information by transforming the BBA into probabilistic function. Furthermore, the BBA can be equally distributed by repeating each BBA among each singleton element. In other words, the pignistic transformation gives an equal probability to elements given a lack of information by building a probability distribution on each element as follows:

(4) $$P_{m}\left(A\right)=\sum_{B\subset \Omega }\frac{\left|A\cap B\right|}{\left|B\right|}m\left(B\right),$$

where |•| denotes the amount of elementary hypotheses of Ω.

### 5.2. Fusion for Tracking Results

Let Θ∈{0,1,Ω} be the frame of discernment for the MOFT, where 0, 1 and Ω represent non-matching, matching and uncertainty. The BBA to tracking results can be defined as follows:

(5) $$\sum_{A\subseteq \Theta }m\left(A\right)=1,$$

where m(A) is a basic belief mass (BBM) representing the part of belief committed to the subset. The conjunctive rule of combination is used to combine the BBA of RGB frames with the BBA of depth frames. We already know that an instance from one modality is corresponding with that from the other modality because 3D point clouds were projected to the image coordinates as a depth image (Section 3). Therefore, we can fuse tracking results generated from different modalities by considering only the final results from the matching networks. Let $m_{D}$ and $m_{R}$ be the BBMs for the tracking results of the depth frames and the RGB frames, respectively. The conjunctive combination $m_{D}⊕m_{R}$ is defined as follows:

(6) $$m_{D}⊕m_{R}\left(A\right)=\sum_{B,C\subseteq \Theta :B\cap C=A}m_{D}\left(B\right)m_{R}\left(C\right),\forall A\subseteq \Theta .$$

The main idea underlying the proposed fusion scheme is the assignment of weights according to the tracking results. To do this, we add a discounting factor to each BBM. The BBM can be discounted when the correctness of a BBM is only valid with a discounting factor. Clearly, the discounting factor α indicates that the degree of trust be applied to the tracking results. The discounting factor for the tracking results of each modality can be defined as follows:

(7) $$\begin{array}{cc} m^{\alpha }\left(A\right)= & (1-\alpha )m(A),\forall A\subseteq \Theta ,A\neq \Theta ,\\ m^{\alpha }\left(\Theta \right)= & \alpha +(1-\alpha )m(\Theta ). \end{array}$$

We set the discounting factor α using the normalized belief functions in [40].

Let us suppose that tracking is independently completed in each modality for the same frames. Before we merge the tracking results, each tracking result should be discounted by their discounting factors. From the given discounted BBMs of the RGB frames $m_{R}^{\alpha_{R}}\left\{{\hat{x}}_{c_{R}}^{t+1}\right\}$ and depth frames $m_{D}^{\alpha_{D}}\left\{{\hat{x}}_{c_{D}}^{t+1}\right\}$, where ${\hat{x}}_{c}^{t+1}$ is the tracked object in each sensor, the joint BBM $m^{\alpha }\left\{{\hat{x}}_{c}^{t+1}\right\}$ is computed as follows:

(8) $$m^{\alpha }\left\{{\hat{x}}_{c}^{t+1}\right\}=m_{R}^{\alpha_{R}}\left\{{\hat{x}}_{c_{R}}^{t+1}\right\}⊕m_{D}^{\alpha_{D}}\left\{{\hat{x}}_{c_{D}}^{t+1}\right\},$$

where α is computed by a linear function and a quadratic function of a minimization program [40]. In this work, we set $\alpha_{R}=0.22$ and $\alpha_{D}=0.31$.

## 6. Structured Fine-Tuning of the Matching Network

**Figure 4:**
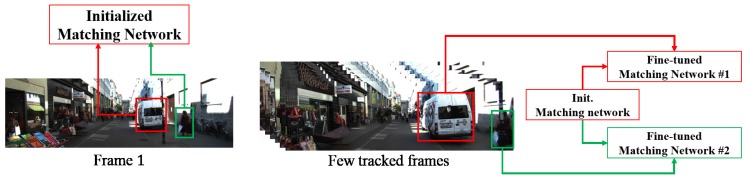
The concept of the structured fine-tuning of target appearance models.

In this section, we describe how to fine-tune the matching network for updating target appearance models and perform tracking using the target appearance models. To build a robust matching network, the initialized matching network trained on external video sequences is fine-tuned after performing tracking on a few frames in a structured model. The fine-tuned matching network can preserve model consistency and become robust to temporally-changed target appearances. At this point, previously fine-tuned matching networks are maintained as paths of the target appearance models. Figure 4 shows an example of our proposed structured fine-tuning of the matching network.

### 6.1. Structured Target Appearance Models

The proposed structured target appearance model is constructed as a hierarchical tree structure by adaptively fine-tuning the matching network. Let S={V,E} be a node for the fine-tuned matching network, where v∈V and (u,v)∈E denote a vertex related to a fine-tuned matching network and a directed edge indicating the path relationship between vertices, respectively. The relationship between two vertices (an edge) is defined as follows:

(9) $$\phi_{E}\left(u,v\right)=\frac{\sum_{f\in \Theta_{F}^{v}}M_{u}\left(x_{i\in n}^{f-1},x_{j\in m}^{f}\right)}{|\Theta_{F}^{v}|},$$

where $\phi_{E}\left(u,v\right)$ denotes the relationship score between vertices *u* and *v*, $\Theta_{F}^{v}$ is a set of consecutive frames on which tracking is performed by using the matching network until the vertex *v* and $M_{u}\left(x_{i\in n}^{f-1},x_{j\in m}^{f}\right)$ is the matching score when $x_{j\in m}^{f}$ is judged as a matched candidate with a previous target object $x_{i\in n}^{f-1}$ by using vertex *u*.

### 6.2. Inference

The target state from a new frame fis inferred by accumulating the relationship scores from multiple fine-tuned matching networks in the form of a structure. Let $X^{f-1}\in \left\{x_{1}^{f-1},x_{2}^{f-1},\ldots ,x_{n}^{f-1}\right\}$ be a set of target objects in frame f−1. The target candidates in frame *f* are $X^{f}\in \left\{x_{1}^{f},x_{2}^{f},\ldots ,x_{m}^{f}\right\}$. The aim of this task is to find the set of most similar pairs *C* among the set $x_{n}^{f-1}$ and $x_{m}^{f}$ as follows:

(10) $${\hat{x}}_{c\in C}^{f}=arg\max_{n}M_{n}\left(x_{i\in n}^{f-1},x_{j\in m}^{f}\right),$$

where $M_{n}$ is a weighted average matching score from the activated fine-tuning path of the *n*-th target object. The activated fine-tuning path of our paper denotes the optimized path that satisfies Equation (3). If a path for a new frame is selected as an activation, the remainder paths are left as deactivated paths, i.e., the remainders are not used ever for the frame. When $V_{\alpha }^{n}\subseteq V$ is the activated fine-tuning path of the *n*-th target appearance model, the weighted average matching score can be measured as follows:

(11) $$M_{n}\left(x_{i\in n}^{f-1},x_{j\in m}^{f}\right)=\sum_{v\in V_{\alpha }^{n}}w_{v\to f}^{n}M_{v}^{n}\left(x_{i\in n}^{f-1},x_{j\in m}^{f}\right),$$

where $M_{v}^{n}\left(x_{i\in n}^{f-1},x_{j\in m}^{f}\right)$ denotes the matching score between $x_{i\in n}^{f-1}$ and $x_{j\in m}^{f}$ corresponding to the vertex *v* of the *n*-th target appearance models, which considers the probability to Class 1 (matching), and $w_{v\to f}^{n}$ is the weight of vertex *v* in frame *f* on a path of the *n*-th target object. If a candidate is not matched with the target object, it is considered as a newly-detected object to track. Further, if a target object has lower matching scores than $\theta_{dis}$ for all candidates, it is considered to be a disappearing object. In this work, we experimentally set $\theta_{dis}$ to 0.6.

To determine weight $w_{v\to f}^{n}$, we consider the reliability of the matching network. This weight is assigned to prevent that fine-tuning is generated on unreliable cases, for which high matching scores are measured despite noisy instances. To measure the reliability of a matching network, the fine-tuning paths, and not in entire paths, are recursively explored as follows:

(12) $$w_{v\to f}^{n}=\frac{\min\left(\phi_{E}\left(P_{v},v\right),w_{P_{v}}^{n}\right)}{\sum_{v\in V_{\alpha }^{n}}\min\left(\phi_{E}\left(P_{v},v\right),w_{P_{v}}^{n}\right)},$$

where $P_{v}$ is the parent node of vertex *v*.

### 6.3. Adaptive Model Update

In the structured target appearance models, a node includes a fine-tuned matching network. We describe an approach to precisely select the fine-tuning path for new training frames.

Let *z* denote a newly-created node to fine-tune the matching network. The matching network is fine-tuned after tracking is completed on 15 consecutive frames without model update, i.e., $|\Theta_{F}^{z}|=15$. The newly fine-tuned matching network has a parent node $P_{z}$ to maximize reliability, which satisfies:

(13) $$P_{z}=arg\max_{v\in V_{\alpha }^{n}}\min\left({\bar{\phi }}_{E},\min\left(\phi_{E}\left(P_{v},v\right),w_{P_{v}}^{n}\right)\right),$$

where ${\bar{\phi }}_{E}$ is the interim edge. Equation (6) can be solved by using a tree searching method, easily. After finding the parent node of the newly-created node *z*, fine-tuning is performed on two sets of frames $|\Theta_{F}^{z}|$ and $|\Theta_{F}^{P_{z}}|$ on only fully-connected layers because fine-tuning all layers including convolutional layers is too expensive to perform and update online. Although the fine-tuning is performed on only fully-connected layers, there is no significant performance degradation as compared to fine-tuning for all of the layers. After the 10th fine-tuning is completed on a path, the earliest node is eliminated when the new fine-tuned matching network is added. Finally, the newly fine-tuned matching network of node *z* is added to $V_{\alpha }^{n}$.

## 7. Experimental Evaluation

In this section, we discuss the evaluations conducted in terms of performance and effectiveness. First, we validate the architectural design and algorithms for the proposed MOFT by comparing it with intra-varied MOFT. Then, we compare it with state-of-the-art trackers on tracking benchmarks.

### 7.1. Dataset and Evaluation Metric

Dataset: We evaluated our proposed method on a project of Karlsruhe Institute of Technology and Toyota Technological Institute (KITTI) tracking [42] and MOT benchmark [43] datasets. We constructed a pair dataset consisting of cropped objects in frames *k* and k+1 from both RGB and depth frames for the training data.

The KITTI tracking dataset contains data captured in driving environments. It consists of 21 training sequences with annotations and also provides various sensor modalities, such as single image, stereo image and 3D point clouds. In our experiments, we used two kinds of paired modalities: (1) RGB frames with depth frames extracted from a stereo camera and (2) RGB frames with depth frames extracted from 3D point clouds. For training, we used 40,000 pairs from 18 training sequences; the remainder was used for evaluations.

We used both the 2015 and 2016 MOT benchmarks (2015: 11 sequences; 2016: seven sequences). Because the MOT benchmark does not provide the depth sequences, we only generated MOFT on RGB frames when evaluations were conducted on the MOT benchmark. For training, we used 32,000 pairs from 15 training sequences; the remainder was used for evaluations.

Evaluation metrics: The following tracking evaluation metrics [44,45] from both benchmarks were utilized: multiple object tracking accuracy (MOTA), multiple object tracking precision (MOTP), mostly tracked targets (MT), mostly lost targets (ML) and the number of ID switches (IDS).

### 7.2. Experimental Setup

Environments: We implemented the proposed method using Caffe [39] and MATLAB on an Intel-Core i7-6700 quad-core 4.0-GHz processor with 64.00 GB of RAM and an NVIDIA GeForce Titan X graphics card with 12 GB of memory for CUDA computations.

Tracking candidates: In this work, the object detector in [36] was used to sample the target candidates. Furthermore, the candidate sampling methods in [11] were used as a comparison model.

Depth frames extraction: We used the adaptive random work method proposed by Lee et al. [46] to extract the depth frames from the stereo camera of the KITTI tracking benchmark. To track objects in 3D point clouds from the KITTI benchmark, we mapped the 3D point clouds into a 2D dense depth map using up-sampling [33].

Data representation: In this work, we used the VGG-16 network [34] pre-trained on the ILSVRC2012 dataset [47] to represent RGB image data. With the same layered architecture, supervision transfer was applied to the depth map for data representation. The scale-level range was set to five levels because the VGG-16 network has five convolutional layers. To classify the scale-level of instances, we used scale-dependent pooling (SDP) [35].

Bounding box regression: If we have used a candidate sampling method or detection strategy, we generated a refinement strategy for the bounding boxes extracted from each frame using the method presented by Girshick et al. [37] to precisely localize the bounding box of target objects. Bounding box regression facilitates accuracy in the tracked target boxes by training four ridge regressors (x,y,w,h), where (x,y) is the center coordinates of the box and (w,h) is the width and height of the box. In our work, the regressors are not updated during testing because of noise.

### 7.3. Evaluation

We evaluated the proposed method by comparing it with intra-varied MOFTs to validate the performance of our architecture choices for MOFT. The matching target experiment was conducted to validate the suitability of the matching between target objects in frame *k* and candidates in frame k+1 for MOT tasks. In the representation architecture experiment, we observed which pre-trained CNNs precisely represent instances and whether adaptively representing instances in accordance with their scale levels is more suitable than representing in a fixed form. The data modality experiment was conducted to show the effectiveness of the proposed fusion tracking method. To show whether the structured fine-tuning update improves the performance of MOFT or not, we compared MOFT with and without fine-tuning in the update experiment. The proposed matching function was designed for application to any unseen target. The generality experiment was conducted to verify this issue. Finally, we compared the proposed MOFT with state-of-the-art trackers.

The basic setting for the proposed method (the row ours in Table 1) was as follows: representation using VGG-16 architectures, adaptive representation according to scale levels of the instance, applying supervision transfer to represent depth data, matching targets in frame *k* with candidates in frame k+1 and the result fusion using BBMs. All of the models, including the proposed model, were evaluated using RGB and depth fusion trackers (except in the data modality and generality experiments). The depth data were extracted from 3D point cloud sequences from the KITTI tracking benchmark dataset. The shown tracking results validated on KITTI benchmark were measured as the average in the entire object classes.

Matching target: To show which target models are suitable for matching with target candidates, we compared ours to a method that matches initialized target objects in the first frame with candidates in the current frame ($model_{1}$). To train the matching function for $model_{1}$, we constructed a dataset in which a pair consisted of a cropped object from the first frame and the corresponding object from the frames remaining in a sequence. As shown in the rows of ours and $model_{1}$ in Table 2, the matching between consecutive frames (ours) can more accurately track the multiple objects than $model_{1}$ in most metrics. Because the target states of the current frame are significantly influenced in the previous frame, noises introduced by temporally-changed states can have a negative effect on the matching function.

Representation architecture: First, we compared the pre-trained network architectures on their ability to represent instances. For a comparison target, we used AlexNet [48] pre-trained on ILSVRC2012 ($model_{2}$) because it is a popular network architecture that comprises smaller layers than VGG-16. As in our proposed model, we divided the scale into five levels for adaptively representing instances on AlexNet. The rows ours and $model_{2}$ in Table 2 show that the representation of instances from the larger network (VGG-16) has better tracking performances on all metrics.

Next, we compared the performances of trackers according to the usages of layers for representation. The following comparison models were used: outputs of conv1−only ($model_{3}$), conv5−only ($model_{4}$), fc7−only ($model_{5}$) and conv5 with fc7 ($model_{6}$) layers from VGG-16. To uniformly feed different sizes of layered representations into our matching network, we applied the sampling scheme described in Section 4.1 into each layered representation. From the results shown in the rows ours and $model_{3,\ldots ,6}$ in Table 2, it is clear that adaptively representing instances according to their scale levels results in more accurate tracking than representing all scale levels of instances in the fixed layers, because information loss is prevented.

Data modality: To observe accuracy differences in the used data modality, we measured the performance when the tracker was generated on each sensor. $model_{7}$ was generated on RGB sequences, and $model_{8}$ and $model_{9}$ were generated on depth sequences extracted from stereo camera and 3D point clouds, respectively. As shown in the rows $model_{7,\ldots ,9}$, MOTA was the highest when tracking was generated on only RGB sequences, whereas the MOTPs of $model_{8}$ and $model_{9}$ were higher than that of $model_{7}$. As a result, ours gave the best performances on all of the metrics compared with $model_{7,\ldots ,9}$. Thus, it is clear that information conflicts generated from modalities can be compensated by combining the tracking results of modalities.

Update: To show that the proposed structured fine-tuning makes MOFT become robust, we compared ours with MOFT without the fine-tuning procedure. As a result, the entire metrics are high for MOFT tracked objects with the structured fine-tuning. As shown in the rows $model_{10}$, however, the performance of MOFT without the structured fine-tuning is not a low.

Generality: This experiment was used to verify that the proposed matching function can be generally applied without references to test sequences. To this end, we evaluated the trackers in two ways: (1) training and testing the matching function on the same dataset and (2) training and testing the matching function on different datasets. This evaluation was conducted on both the MOT [43] and KITTI [42] benchmark datasets. Because the MOT benchmark does not include depth data, the trackers only tracked objects on RGB sequences in this experiment. Table 3 shows the dataset used for training and testing and the resulting performances. The first row in Table 3 is the same as $model_{7}$ in Table 1 and Table 2. As a result, although MOFTs for which training and testing are performed on the same dataset can track objects more accurately than other cases, there was no significant performance degradation when comparing the first and third rows or the second and fourth rows.

State-of-the-art comparisons: One of the advantages of MOFT stated above is that it is robust to distortion factors because of a matching function trained on external video sequences offline. Further, information conflicts can be compensated by employing a modality fusion scheme.

Table 4 shows the tracking performances on the MOT16 dataset. Table 5 and Table 6 show the tracking performances on the KITTI tracking benchmark. On the MOT benchmark, MOFT provides the best performance in terms of MOTA and MT. On the KITTI benchmark, MCMOT-CPT, which uses a general CNN detector, has the higher performances on the car category, while the MOFT has the higher performance on the pedestrian category. Generally, the instances of pedestrians have a low resolution while an instance including car has an adequate resolution, which can avoid information losses raised from passing many layers of CNNs. In other words, if an instance including a pedestrian was passed in many layers of CNNs, information losses can be easily introduced. Therefore, the MOFT can track multiple objects without category-dependence because the MOFT adaptively represents instances according to their sizes. Further, MOFT achieved state-of-the-art performances on the other metrics.

**Figure 5:**
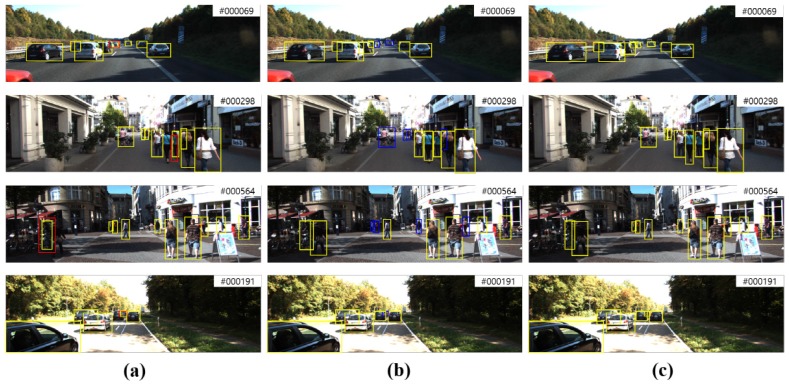
Comparison of tracked targets on: (**a**) RGB frames; (**b**) depth frames extracted from 3D point clouds; and (**c**) MOFT. Each box indicates the following: yellow box: correctly-tracked objects; red box: shifted objects; blue box: missed objects.

Qualitative results: MOFT was only generated on the KITTI benchmark dataset to qualitatively evaluate the proposed tracker because the MOT benchmark does not include depth data. Figure 5a,b depicts the tracked targets of each tracker on RGB and depth (from 3D point clouds) frames, respectively. Figure 5c shows the tracked targets of MOFT. It can be seen that, whereas the bounding boxes are shifted to similar objects in the RGB tracker, the depth tracker cannot precisely track the distant targets. MOFT compensates the limitations of each modality. As shown in Figure 5, the tracking failures are overcome.

**Figure 6:**
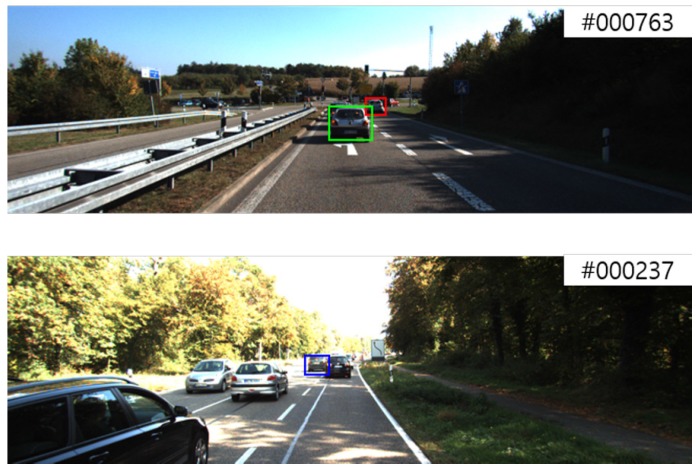
Failure cases of MOFT. Each box indicates the following: green box: ground truth; red box: shifted object; blue box: missed object.

Although a modality fusion procedure is applied with discounting factors in MOFT, tracking failures from the failures of each modality still occurred. On the top of Figure 6, bounding box shifting and missing between similar targets can be observed. This may be as a result of the influence of the RGB tracker. On the bottom of Figure 6, distant objects are considered as disappearing objects. Even though they maintain tracking-available sizes for the RGB tracker, they may be overlooked in the depth tracker.

## 8. Conclusions

In this paper, we proposed a multiple objects fusion tracker called MOFT. We demonstrated that we can train a matching function regardless of disturbing factors using a matching network. In addition, because each instance has a different scale level, we adaptively represent instances from layered representations of the pre-trained CNN. Further, to compensate tracking failures occurring in each modality, we proposed a fusion tracker based on combining BBMs. Our fusion tracker fuses the tracking results of each modality at the decision level to prevent information conflicts introduced by feature-level fusion schemes. MOFT was verified as achieving better results than conventional multiple object trackers on the KITTI and MOT benchmarks.

In future work, we will plan to extend MOFT to combine various modalities. We will also propose methods for real-time computation.

## Figures and Tables

**Table 1:** Comparison models used to evaluate the proposed MOFT. Depth (PC) and Depth (stereo) denote depth frames extracted from 3D point clouds and stereo vision, respectively. “Init.” indicates the initialized target. w and w/o of the “update” column mean that the proposed fine-tuning method was used for MOFT (w) or not (w/o), respectively.

Tracker	Matching Target	Representation	Representation Usage	Modality	Update
ours	k&k+1	VGG-16	Adaptively	RGB + Depth (PC)	w
$model_{1}$	Init.&k	VGG-16	Adaptively	RGB + Depth (PC)	w
$model_{2}$	k&k+1	AlexNet	Adaptively	RGB + Depth (PC)	w
$model_{3}$	k&k+1	VGG-16	conv1−only	RGB + Depth (PC)	w
$model_{4}$	k&k+1	VGG-16	conv5−only	RGB + Depth (PC)	w
$model_{5}$	k&k+1	VGG-16	fc7−only	RGB + Depth (PC)	w
$model_{6}$	k&k+1	VGG-16	conv5+fc7	RGB + Depth (PC)	w
$model_{7}$	k&k+1	VGG-16	Adaptively	RGB	w
$model_{8}$	k&k+1	VGG-16	Adaptively	Depth (PC)	w
$model_{9}$	k&k+1	VGG-16	Adaptively	Depth (stereo)	w
$model_{10}$	k&k+1	VGG-16	Adaptively	RGB + Depth (PC)	w/o

**Table 2:** Comparison of the proposed MOFT with design-varied trackers on testing sequences. The best and second-best scores are boldfaced and underlined, respectively. The direction of the arrows indicates the direction of better performances; multiple object tracking accuracy (MOTA), multiple object tracking precision (MOTP), mostly tracked targets (MT), mostly lost targets (ML) and the number of ID switches (IDS).

Tracker	MOTA↑	MOTP↑	MT↑	ML↓	IDS↓
ours	**66.38**%	**78.95**%	**30.89**%	**21.79**%	**7**
$model_{1}$	60.22%	78.83%	23.24%	30.44%	27
$model_{2}$	62.42%	69.25%	24.58%	31.57%	20
$model_{3}$	59.14%	74.22%	24.97%	29.12%	22
$model_{4}$	59.46%	73.39%	26.14%	27.41%	29
$model_{5}$	48.49%	61.44%	18.62%	32.01%	11
$model_{6}$	64.21%	77.61%	27.55%	24.88%	29
$model_{7}$	61.51%	63.20%	26.04%	33.63%	31
$model_{8}$	60.55%	68.24%	26.88%	34.58%	30
$model_{9}$	60.48%	66.91%	27.43%	28.29%	26
$model_{10}$	63.47%	77.79%	28.81%	23.33%	14

**Table 3:** Comparison of the performances according to training and testing datasets. **M** and **K** are MOT15 and 16 and KITTI benchmarks, respectively. **A**→**B** of the model indicates that the matching network was trained on dataset **A**, whereas the testing was generated on dataset **B**. The direction of the arrows indicates the direction of better performances; multiple object tracking accuracy (MOTA), multiple object tracking precision (MOTP), mostly tracked targets (MT), mostly lost targets (ML) and the number of ID switches (IDS).

Tracker	MOTA↑	MOTP↑	MT↑	ML↓	IDS↓
$ours_{K\to K}$	61.51%	63.20%	26.04%	33.63%	31
$ours_{M\to M}$	46.88%	77.24%	18.92%	46.54%	41
$ours_{M\to K}$	60.11%	61.09%	22.23%	33.98%	33
$ours_{K\to M}$	45.92%	77.16%	17.99%	45.98%	43

**Table 4:** Comparison of the proposed MOFT with previous trackers on the MOT16 [49] benchmark dataset. The boldfaced and underlined scores indicate the best and second-best scores, respectively. The direction of the arrows indicates the direction of better performances; multiple object tracking accuracy (MOTA), multiple object tracking precision (MOTP), mostly tracked targets (MT), and mostly lost targets (ML).

Tracker	MOTA↑	MOTP↑	MT↑	ML↓
TBD [50]	33.7%	**78.5**%	7.2%	54.2%
LTTSC-CRF [51]	37.6%	75.9%	9.6%	55.2%
OVBT [52]	38.4%	75.4%	7.5%	47.3%
EAMTT-pub [53]	38.8%	75.1%	7.9%	49.1%
LINF1 [54]	41.0%	74.8%	11.6%	51.3%
MHT-DAM [55]	42.9%	76.6%	13.6%	46.9%
oICF [56]	43.2%	74.3%	11.3%	48.5%
JMC [57]	46.3%	75.7%	15.5%	**39.7**%
NOMT [58]	46.4%	76.6%	18.3%	41.4%
ours	**46.78**%	77.95%	**19.41**%	45.34%

**Table 5:** Comparison of the proposed MOFT with previous trackers on the KITTI benchmark dataset [59]. This evaluation was validated on the car category. The boldfaced and underlined scores indicate the best and second-best scores, respectively. The direction of the arrows indicates the direction of better performances; multiple object tracking accuracy (MOTA), multiple object tracking precision (MOTP), mostly tracked targets (MT), and mostly lost targets (ML).

Tracker	MOTA↑	MOTP↑	MT↑	ML↓
SCEA [60]	51.30%	78.84%	26.22%	26.22%
TBD [50]	49.52%	78.35%	20.27%	32.16%
NOMT [58]	55.87%	78.17%	**39.94**%	25.46%
CEM [61]	44.31%	77.11%	19.51%	31.40%
DCO [62]	28.72%	74.36%	15.24%	30.79%
mbodSSP [63]	48.00%	77.52%	22.10%	27.44%
HM [64]	41.47%	78.34%	11.59%	39.33%
DP-MCF [65]	35.72%	78.41%	16.92%	35.67%
MCMOT-CPD [66]	72.11%	82.13%	52.13%	11.43%
ours	**65.48**%	**79.27**%	32.61%	**18.41**%

**Table 6:** Comparison of the proposed MOFT with previous trackers on the KITTI benchmark dataset [59]. This evaluation was validated on the pedestrian category. The boldfaced and underlined scores indicate the best and second-best scores, respectively. The direction of the arrows indicates the direction of better performances; multiple object tracking accuracy (MOTA), multiple object tracking precision (MOTP), mostly tracked targets (MT), and mostly lost targets (ML).

Tracker	MOTA↑	MOTP↑	MT↑	ML↓
SCEA [60]	26.02%	68.45%	9.62%	47.08%
NOMT-HM [58]	17.26%	67.99%	14.09%	50.52%
NOMT [58]	25.55%	67.75%	17.53%	42.61%
CEM [61]	18.18%	68.48%	8.93%	51.89%
RMOT [67]	25.47%	68.06%	13.06%	47.42%
MCMOT-CPD [66]	40.50%	**72.44**%	20.62%	**34.36**%
ours	**44.87**%	70.55%	**24.60**%	37.92%
